# High-Temperature Storage Testing of ACF Attached Sensor Structures

**DOI:** 10.3390/ma8125455

**Published:** 2015-12-10

**Authors:** Sanna Lahokallio, Maija Hoikkanen, Jyrki Vuorinen, Laura Frisk

**Affiliations:** 1 Department of Electrical Engineering, Tampere University of Technology, P.O. Box 692, Tampere 33101, Finland; laura.frisk@tut.fi; 2 Department of Materials Science, Tampere University of Technology, P.O. Box 589, Tampere 33101, Finland; maija.hoikkanen@gmail.com (M.H.); jyrki.vuorinen@tut.fi (J.V.)

**Keywords:** anisotropically conductive films, organic printed circuit board materials, reliability testing, high temperature testing, material characterization, failure analysis

## Abstract

Several electronic applications must withstand elevated temperatures during their lifetime. Materials and packages for use in high temperatures have been designed, but they are often very expensive, have limited compatibility with materials, structures, and processing techniques, and are less readily available than traditional materials. Thus, there is an increasing interest in using low-cost polymer materials in high temperature applications. This paper studies the performance and reliability of sensor structures attached with anisotropically conductive adhesive film (ACF) on two different organic printed circuit board (PCB) materials: FR-4 and Rogers. The test samples were aged at 200 °C and 240 °C and monitored electrically during the test. Material characterization techniques were also used to analyze the behavior of the materials. Rogers PCB was observed to be more stable at high temperatures in spite of degradation observed, especially during the first 120 h of aging. The electrical reliability was very good with Rogers. At 200 °C, the failures occurred after 2000 h of testing, and even at 240 °C the interconnections were functional for 400 h. The study indicates that, even though these ACFs were not designed for use in high temperatures, with stable PCB material they are promising interconnection materials at elevated temperatures, especially at 200 °C. However, the fragility of the structure due to material degradation may cause reliability problems in long-term high temperature exposure.

## 1. Introduction

Electronics products are used increasingly in demanding environments, which may cause reliability problems. One such condition is high temperature, which may significantly exceed the typically accepted upper temperature limit of 125 °C [1,2]. When the temperature is increased, the functional limits of traditionally used package and attachment materials are often exceeded, imposing challenges and restrictions on material choices and structures. It is vital that the chosen packaging materials and structures are reliable for use in temperatures but are also cost-effective and easily available, making the use of inexpensive, commonly used materials interesting for elevated temperature applications.

Typically, metallic or ceramic materials are used in the packaging of high-temperature electronics. This is because of their excellent stability. However, metals and ceramics are more expensive and less versatile than polymer materials, and consequently, there is an increasing interest in using low-cost polymer materials in high-temperature applications. According to the literature, traditional polymer packages and interconnection materials should not be used above 150 °C due to detrimental loss of electrical and mechanical properties occurring at higher temperatures [1,3]. The important parameters for polymers at high temperatures include glass transition temperature (*T*_g_), melting temperature (*T*_m_), and coefficient of thermal expansion (CTE). For thermoplastic materials, either *T*_g_ or *T*_m_ is the upper limit of use, since the materials melt above these temperatures. For thermoset polymers, *T*_g_ is the critical temperature. However, thermoset polymers do not melt at *T*_g_ and thus can be used at temperatures above their *T*_g_. On the other hand, when the *T*_g_ of a thermoset is exceeded, its mechanical properties tend to deteriorate significantly, making the material potentially unreliable. Additionally, above *T*_g_ the CTE typically increases rapidly, causing considerable thermomechanical stresses on the structures. Therefore, cost-effective polymer materials having sufficiently high *T*_g_ and *T*_m_ values are needed for high-temperature electronics. In addition to the thermal parameters, the long-term stability of polymers is critical. Long term exposure to temperatures below the thermal limits of a polymer may still degrade the material, thus making it unreliable.

As mentioned above, ceramic substrates, high- or low-temperature co-fired ceramic (HTCC, LTCC) boards or diamond substrates are often recommended for high-temperature use [4,5,6]. They are, however, much more expensive than organic printed circuit boards (PCBs) [6], and may have problems with mechanical and electrical integrity and resistivity [4]. They are also less readily available and their production requires special equipment. There are also interesting organic PCB materials available for high-temperature use, however. Polyimides (PI) are frequently used substrate materials due to their excellent thermal resistance [7], and they can be used at temperatures above 300 °C [8]. The metallization is, however, typically attached to the PI with an adhesive layer having inferior properties, thereby impairing the reliability of the substrate [7,9,10]. Fluoropolymers such as PTFE are also interesting high-temperature substrate materials, but they are difficult to process and prone to adhesion problems [7,11]. Additionally, FR-4 PCBs are available with higher *T*_g_ values. It is also possible to use hybrid boards made of polymers and ceramics [2]. They can often be processed with the same process as FR-4 and the costs are much lower than with pure ceramic boards [11]. One example of these PCBs is hydrocarbon-ceramic laminates, also known by the trade name Rogers.

High-temperature soldering materials, such as Pb_2_Sn_2.5_Ag, containing harmful lead [12], and hard solders, for example Au/Si and Au/Ge [1], have been developed for interconnections requiring high temperature resistance. However, fewer options are available for components that cannot be soldered. Electrically conductive adhesives (ECA) are an interesting option for such interconnections. ECAs are polymer adhesives to which conductive particles have been added. There are two types of ECAs available: isotropic conductive adhesives (ICA) and anisotropic conductive adhesives (ACA). ICAs have a large number of conductive particles and they can be used similarly to solders. High-temperature ICAs have been developed and are commercially available. ACAs have smaller concentration of particles and are especially well suited for flip chip attachments. No high temperature ACAs are commercially available.

Anisotropically conductive films (ACFs) are ACAs in film form and are commonly used in flip chip attachments. They can be used with a wide range of different substrates, dies, and metallization materials. ACF flip chip applications do not need additional underfill, and they have a fine pitch capability [13]. Anisotropically conductive adhesives are used, for example, in radio-frequency identification (RFID) tags [14], in liquid crystal displays (LCD) [15], and memory chips [16] in mobile applications as well as in various fine-pitch applications [17]. ACFs are also suitable for many different sensor applications. The polymer matrix in ACFs provides mechanical support and acts as an insulator in *x* and *y* directions. A unidirectional conductivity is formed in the *z*-direction only after the attachment process, in which some of the particles are trapped between the contact surfaces attached [18]. Thermoset epoxy is the most widely used matrix material [19] and particles are typically gold-coated polymer particles or metal particles, often made of nickel [16].

Due to its cross-linked structure, the thermoset epoxy matrix in ACFs is partly able to maintain its strength at temperatures above its *T*_g_. Therefore, the actual temperature usage limit for thermoset ACFs can be higher than the attachment temperature, unlike solder materials. Exceeding the *T*_g_ may still cause considerable reliability concerns, such as decreased stiffness [20] and post-curing, leading to brittleness [21,22]. Both promising and discouraging results for using polymer-based adhesive interconnection materials have been reported, but the field is not widely studied [2,22,23,24,25,26,27,28,29,30,31,32].

In our earlier studies the reliability results have been promising for ACF attachments with PI substrates [29,30,31,32]. However, problems caused by oxidation and degradation were found for the PI substrate. The aim of this paper was to study the effect of substrate on the high-temperature reliability of a flip chip attached temperature sensor package with ACF interconnections. Two different organic substrates were studied from electrical and mechanical points of view. The aim was to study substrate materials that can be processed with normal printed circuit board processes, and to compare the results to a commonly used FR-4 PCB. The sensor component studied was a resistance-based temperature sensor designed for use in challenging environments. The sensors were attached with ACF to rigid Rogers and FR-4 PCBs using flip chip technique. Electrical reliability was studied by measuring the resistances of the test samples using continuous real-time measurements during aging at 200 °C and 240 °C in order to observe the occurrence of failures during the tests. Mechanical reliability was studied with shear testing. Degradation behavior was studied with several material characterization techniques. The failure mechanisms were studied with a scanning electron microscope (SEM).

## 2. Experimental

### 2.1. Test Sample

The test structure used included temperature sensors attached onto a substrate with ACF material. The test chip used was a temperature sensor manufactured on an aluminum oxide substrate. The sensor chip was 3.21 mm × 3.26 mm in size with a thickness of 400 μm. Four contact areas of 500 μm in diameter and with a pitch of 830 μm were fabricated on the sensor. The contact areas were not bumped, but coated with less than 1 μm thick Ni/Au layer, providing good contact surface to the ACF attachment. The functionality of the sensor was based on the contact resistance changes in a specific structure on the aluminum oxide substrate.

Two different PCB materials were used, namely a glass-reinforced laminate made of hydrocarbon and ceramic (Rogers) and a glass-reinforced epoxy laminate (FR-4). The layouts were similar for both PCB materials. The PCBs were single-sided. The thicknesses of the PCBs were 0.8 mm (Rogers) and 1 mm (FR-4). The copper wirings on the Rogers PCB had a thickness of 25 μm and were coated with 7 μm thick Ni/Au plating. The copper wirings on the FR-4 PCB had a thickness of 30 μm and were coated with 3 μm thick Ni/Au plating. The PCB was 75 mm × 35 mm in size, having four contact areas for sensor attachments fabricated onto it and each contact area had four pads for the sensor attachments.

Two different ACFs were used to attach the sensor chips onto the substrates. Both adhesives had similar thermoset polymer matrices but different particle types. ACF 1 had gold-coated polymer particles, whereas ACF2 had gold-coated nickel particles. Both ACFs were commercial products from the same manufacturer. They were designed for use in electronics and flip chip applications and not specifically designed for use at high temperatures. The ACFs were chosen on the basis of earlier studies [29,30,31,32]. Table 1 presents particle sizes and filler types as well as the film thicknesses of the ACFs. The *T*_g_, decomposition temperature (*T*_d_), CTE, and modulus values for the ACFs according to their data sheets are also shown. *T*_g_ and modulus values for ACF1 are also shown in brackets according to the results shown in [30].

Sensor chips were attached onto the substrates using flip chip technique. The ACAs were attached with a Toray FC-1000 flip chip bonder. The ACFs were first cut to size and placed on the substrate pads. Then, the adhesive was pre-bonded with flip chip bonder using moderate heat and light pressure for a short period of time. The final bonding was done according to the bonding parameters recommended by the ECA manufacturers, using a bonding temperature of 210 °C for 20 s with pressure of 100 MPa.

**Table 1:** Properties of the ACFs studied. ACA, Anisotropic conductive adhesive; *T*_g_, Glass transition temperature; *T*_d_, Decomposition temperature; CTE, Coefficient of Thermal Expansion.

ACA	Particle Type	Particle Size (μm)	Film Thickness (μm)	*T*_g_ Datasheet (°C)	*T*_d_ (°C)	CTE (ppm)	Modulus (GPa)
ACF1	Polymer with Au coating	4	30	112 (101)	330	40/561	2.6(4)
ACF2	NiAu	8	40	113	332	39/552	2.3

### 2.2. Test Methods

The behavior of the ACF interconnections and the substrates at high temperatures was studied by two thermal storage tests at constant temperatures of 200 °C and 240 °C. During testing the temperature was raised from ambient to testing temperature in two hours, after which the testing was continued at a constant temperature. Testing was conducted in a Binder M240 thermal chamber. It was not possible to remove individual samples from the thermal chamber after they had failed. Therefore, all the samples were tested for the same period of time regardless of their failures. Duration of testing at 200 °C was 3000 h for both substrates, and 654 h and 1000 h at 240 °C for Rogers and FR-4 PCBs respectively. The number of test samples for each test lot in all tests was 7–8 attachments with each ACF. The contact resistances of the samples were measured in real-time during testing (Section 2.3). In addition to the basic series in which contact resistances were measured in real-time during the test, additional samples were tested without real-time measurements and these samples were removed from the test after certain periods of time, namely 480 h, 960 h, 1440 h, 1920 h, and 2400 h, respectively, for the samples tested at 200 °C, and for 22 h, 60 h, 124 h, and 500 h for the samples tested at 240 °C.

After testing, the failure data was analyzed with cumulative distribution functions (CDF). For failure analysis, cross-sections of the test samples were prepared before and after testing. The analysis of the cross-sections was conducted with an optical microscope and a scanning electron microscope (SEM).

### 2.3. Resistance Measurement

The resistance values of the interconnections and the sensors were used to study the functionality of the packages. For each sensor, the resistance was measured between two pads on the sensor. These pads were connected through a temperature sensor structure forming a simple daisy chain structure. The sensor structure had a relatively high resistance value. Consequently, the resistance value for the daisy chain structure included the contact resistances of the ACF interconnections, the resistance of the temperature sensor, and the resistance of the tracks on the substrates, and was relatively high. However, the resistance of the sensor structure was known to be stable at these temperatures and to change only according to the temperature increase it was designed for. Therefore, other changes seen in the daisy chain resistance could be assumed to be caused by changes either in the ACF interconnections or in the substrates.

To study the changes in the daisy chain resistance values during testing, the resistances of the test samples were measured using continuous real-time measurements with a data logger system. Each chip had its own measurement channel. A constant, stable current was fed separately through shunt resistors to the measured channels and the voltage over the chip structure was individually measured for each channel. Thus, any rise in the measurement voltage indicated an increase in the sample resistance. As open joints were formed on the test samples, the measured voltage rose to the supply voltage of approximately 5 V. The voltage was measured every 10 s during all tests.

Two different failure limits were used to analyze failure times because the samples were highly sensitive to resistance changes. At 200 °C, the test samples were considered to have failed when the voltage across the chip structure increased to 0.85 V (206 Ω) and to 1 V (256 Ω). These corresponded to increases of 5% and 30% in daisy chain resistance. At 240 °C, the test samples were considered to have failed when the voltage across the chip structure increased to 0.93 V (228 Ω) and to 1 V (256 Ω). These corresponded to increases of 5% and 15% in daisy chain resistance. Furthermore, samples were also considered to have failed if the resistance decreased rapidly, indicating the formation of a short circuit.

### 2.4. Material Characterization

The thermo-oxidative resistance and decomposition of the PCB materials was studied for thermal stability and degradation behavior with simultaneous thermal analysis (STA), a combination of thermogravimetric (TGA) and differential thermal analysis (DTA). The 10–15 mg specimens of cured adhesives were heated from 30 °C to 950 °C at a rate of 10 °C/min under artificial air flow of 20 mL/min with a Perkin Elmer (Waltham, MA, USA) STA 6000, mimicking the degradation conditions in air.

Further thermal analysis was conducted using differential scanning calorimetry (DSC) for the materials before and after aging. For this analysis, the materials were aged in a laboratory oven at 200 °C and 240 °C for 120 h, 480 h, and 1920 h. Mettler Toledo (Greifensee, Switzerland) DSC 1 STARe system was used for the DSC measurements. The measurements were conducted between 40 °C and 300 °C in nitrogen atmosphere with a temperature change rate of 10 K/min. The size of the samples varied between 13 mg and 14 mg for Rogers samples and between 14 and 17 mg for FR-4, except for the sample aged at 240 °C for 1920 h, which had a weight of 11 mg. The same materials were also studied using Fourier transform infrared spectroscopy (FTIR). The FTIR spectra were measured using a Thermo Fisher Scientific (Waltham, MA, USA) Nicolet iS10 FTIR with diamond Attenuated Total Reflectance (ATR) system.

### 2.5. Shear Testing

The mechanical properties of the sensor attachments were studied with shear testing. Shear testing was conducted for the test samples with the Rogers PCB only, and the effect of aging was studied by aging the samples for certain periods of time prior to testing; the results were compared to non-aged samples. The aging times at 200 °C and 240 °C were 30 h, 60 h, 120 h, 480 h, and 1920 h. Eight samples were tested from each aging and temperature series.

Shear testing was conducted with a Nordson Dage (Aylesbury, Buckinghamshire, UK) series 4000 bond tester using the DS100 cartridge tool. The substrate was held still while the sensor chips were pushed from one side with the tool. The maximal force needed to separate the sensor chip from the substrate was measured. The speed used with the shear tool was 400 μm/s and the tool was located 50 μm above the substrate surface during testing.

## 3. Results

### 3.1. Materials Characterization

#### 3.1.1. TGA and DTA Results

The stability and degradation of the PCB materials were studied with TGA and DTA. Table 2 shows the analyzed results from the TGA measurements. Onset temperature corresponds to 1.0% organic mass loss. It can be calculated with Equation (1):

(1) $$Onset=m_{i}-\left(m_{i}-m_{f}\right)\times 0.01$$

**Figure 1:**
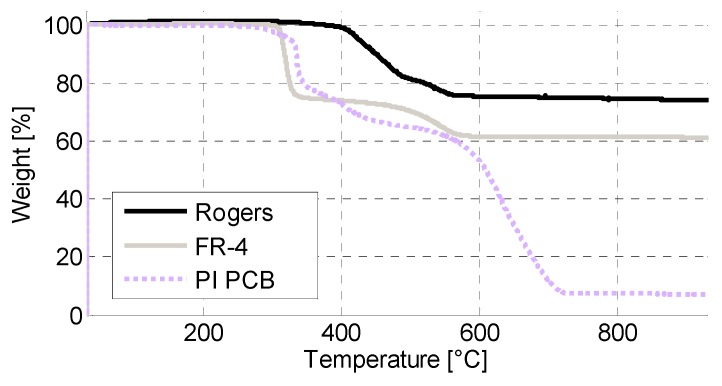
Thermogravimetric analysis results for PCB materials.

In the equation, *m*_i_ is the initial mass of the sample and *m*_f_ is the residual mass of the sample at 930 °C, which can also be used to estimate the fractions of non-oxidizing fillers in the compounds. Weight loss at 240 °C was calculated by comparing *m*_i_ and the sample mass at 240 °C. Table 2 also shows the main steps for mass losses as well as the residual fraction between *m*_i_ and *m*_f_. The main mass loss steps are also shown in Figure 1. The results are compared to PI PCB used in similar studies [30].

**Table 2:** Results from TGA measurements. *m*_i_ is the initial mass of the sample and *m*_f_ is the residual mass of the sample at 930 °C. PCB, Printed Circuit Board; PI, Polyimide.

Material	Onset [°C]	Weight Loss at 240 °C [%]	Step 1		Step 2		Step 3		Residual Fraction (*m*_f_/*m*_i_) [%]
			Temperature Range [°C]	Δ*m* [%]	Temperature Range [°C]	Δ*m* [%]	Temperature Range [°C]	Δ*m* [%]	
Rogers PCB	387.1	0	–	–	382–505	18.8	505–577	5.5	74.1
FR-4 PCB	305.8	0	265–379	26.1	379–597	12.6	–	–	61.3
PI PCB	277.8	0.3	189–346	20.4	346–491	14.5	491–729	57.8	7.2

The DTA signal was used as an additional tool to separate sequential decomposition steps. Table 3 shows the endothermic peaks relative to original masses and corresponding reaction enthalpies. Peaks are listed based on the regions in TGA measurements. Peaks and reaction enthalpies could not be determined for Rogers PCB as no clear peaks were seen. The results for FR-4 are compared to PI PCB used in similar studies [30].

**Table 3:** Results from DTA measurements.

Material	Step 1		Step 2		Step 3	
	Peak [°C]	Δ*H* [J/g]	Peak [°C]	Δ*H* [J/g]	Peak [°C]	Δ*H* [J/g]
FR-4	S, 313	S, −50	–	–	539	−799
PI PCB	S, 334	S, −158	–	–	632 and 698 (double)	−1748

S = small peak, not quantified.

Both Rogers and FR-4 PCBs were shown to be stable at the aging temperatures used. No weight loss could be seen at 200 °C or 240 °C (Figure 1). A slight increase in weight at the start of the run was seen for both materials, indicating oxidation of the materials. Onset occurred for both materials above 300 °C (Table 2). For FR-4, a drastic weight loss occurred above 300 °C, as shown in Figure 1. Rogers PCB was more stable with significant weight loss occurring above 400 °C. The residual fraction after testing was very high for both materials (Table 2). Both PCB materials were clearly more stable than PI PCB. According to the DTA results, however, PI PCB had endothermic transitions above the transitions of FR-4 (Table 3).

#### 3.1.2. DSC Results

The DSC measurements of the FR-4 samples before testing showed a *T*_g_ range of 123 °C–140 °C and a midpoint of 133 °C. However, after the first run up to 300 °C, changes had occurred in the material, and during the second run, a lower *T*_g_ value with a midpoint of 94 °C was seen. The *T*_g_ value of the first measurement was typical for FR-4 materials and therefore the reduction indicated problems with the stability at temperatures below 300 °C, although TGA measurements had not shown degradation below this temperature. No *T*_g_ was seen in the aged samples, which indicated that the material had already degraded after 120 h at 200 °C. Yuan and Packowski have reported a marked reduction in the *T*_g_ value of FR-4 materials occurring in 2 h at 240 °C, showing that the stability of FR-4 may already be poor during a lengthy exposure at relatively low temperatures [33].

**Figure 2:**
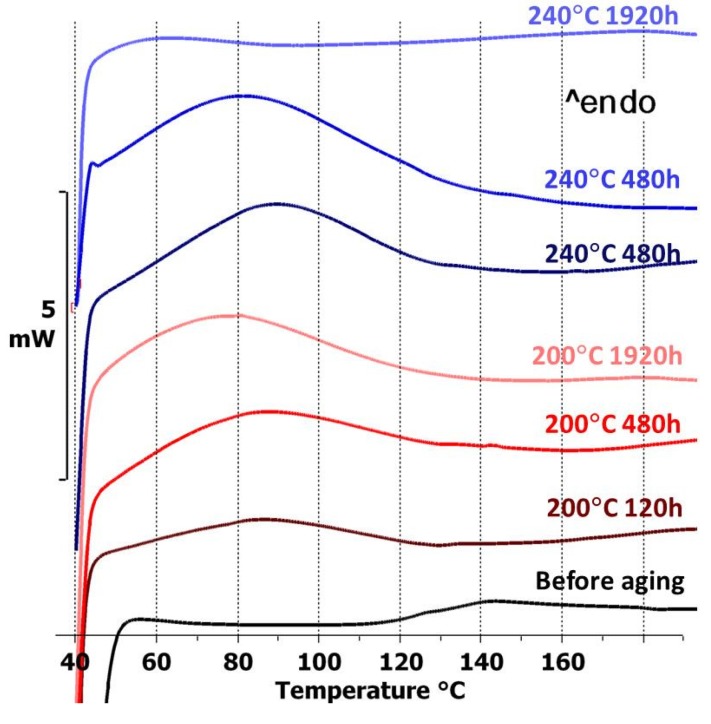
DSC measurements for the FR-4 test board before and after testing.

At the beginning of the first run, each aged FR-4 sample had a wide endothermic peak, except the one aged at 240 °C for 1920 h. This peak was assumed to be caused by evaporation of water from the sample. Figure 2 illustrates the first runs for the FR-4 DSC measurements. The weights of the measured samples were quite similar. As can be seen, the endothermic peaks became more pronounced with increasing testing time at 200 °C. Additionally, considerably higher peaks were seen with the test samples aged at 240 °C. During aging, the organic part of the FR-4 material had lost its structure and turned into a dark brown powdery substance when the boards were cut for the DSC measurements. It was assumed that this change made the material more vulnerable to water absorption, which caused the peaks seen in the DSC measurements. The worse the degradation was, the higher the water evaporation peak seemed to be. However, after the very long exposure at 240 °C, the FR-4 seemed have lost most of its organic part and consisted mainly of glass fiber cloth. This changed its behavior and no water absorption peak was seen.

No peaks or *T*_g_ were seen with the Rogers material before aging. After aging, an endothermic peak related to water absorption similar to that in FR-4 was found. However, this peak was seen only with the samples aged for 1920 h at 200 °C and at 240 °C for 480 h and 1920 h. Furthermore, the peaks were considerably lower than those seen with FR-4. As with FR-4, these peaks were assumed to indicate the degradation of the Rogers material with prolonged exposure to high temperature. However, the change in Rogers was dramatically smaller than in FR-4, indicating considerably better stability for Rogers.

#### 3.1.3. FTIR Results

**Figure 3:**
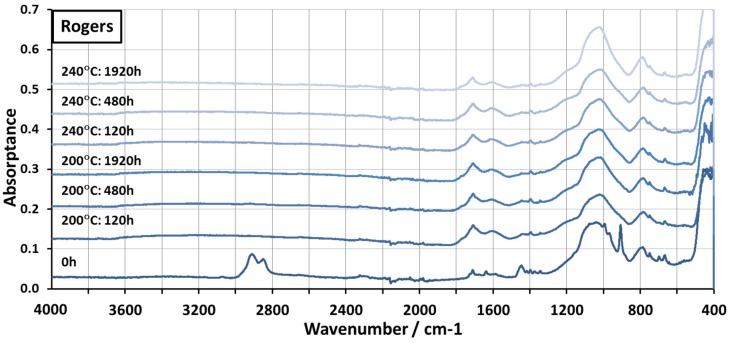
FTIR spectra of the Rogers PCB aged at 200 °C and 240 °C.

Figure 3 represents the FTIR spectra for a Rogers PCB aged at 200 °C and 240 °C. The exact composition of the Rogers material was unknown, but according to the manufacturer, its matrix was composed of hydrocarbon and ceramic. Strong peaks were seen in the samples before testing in the area between 3200 cm^−1^ and 2800 cm^−1^, which are most likely related to aliphatic –CH_3_ and –CH_2_– groups [34]. Additionally, clear peaks were seen at 1711 cm^−1^, 1638 cm^−1^, 1446 cm^−1^, 1041 cm^−1^, 994 cm^−1^, 907 cm^−1^, and 786 cm^−1^. It was not clear whether these peaks were related to the ceramic or hydrocarbon part of the composite. Most of the peaks seen in the non-aged material already disappeared after 120 h of aging at 200 °C. Very little change was seen after this. Two new strong and wide peaks at 1709 cm^−1^ and 1604 cm^−1^ formed in the aged samples. Mostly, the changes seen in the samples aged at 240 °C were very similar to those seen in the samples aged at 200 °C: the changes seen occurred during the first 120 h and the materials seemed to be stable thereafter.

It was assumed that most of the changes seen in the spectra were caused by the oxidation of the hydrocarbon due to the high temperature. During testing, the surface of the material turned from white to brown, showing that changes had occurred. However, after 120 h, the material seemed to be stable and no further changes were seen. The aliphatic peaks between 3200 cm^−1^ and 2800 cm^−1^ disappeared completely, which indicated that these groups had already reacted during the 120 h. The appearance of the peak at 1710 cm^−1^ indicated the formation of carbonyl groups in the material and this supported the assumption that the aliphatic groups had oxidized [34]. Formation of peaks due to thermal oxidation of polyolefins in this area has been reported before [35,36,37].

**Figure 4:**
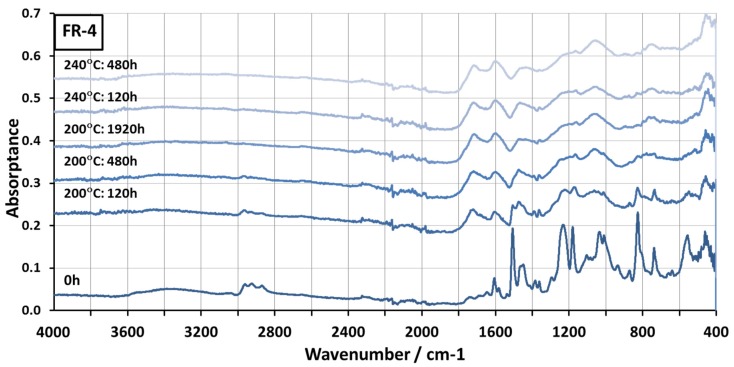
FTIR spectra of the High-*T*_g_ FR-4 PCB aged at 200 °C and at 240 °C.

The FTIR spectra for FR-4 aged at 200 °C and 240 °C are shown in Figure 4. The matrix of FR-4 was assumed to be based on bifunctional diglycidyl ester of bisphenol A (DGEBA) or to have relatively similar chemistry. DGEBA is a commonly used material in the epoxies of PCBs [38,39]. The compositions of the curing agent and flame retardants were unknown. Several peaks were seen in the spectrum before aging. The wide peak at 3424 cm^−1^ was likely caused by the hydroxyl group, which forms when the oxirane structure of epoxy reacts during the curing process [40]. The peaks at 2963 cm^−1^, 2926 cm^−1^, and 2870 cm^−1^ were assumed to be caused by aliphatic –CH_3_ and –CH_2_– groups [34]. The peaks at 1606 cm^−1^, 1507 cm^−1^, and 825 cm^−1^ were assumed to be caused by the aromatic ring, and the peaks at 1234 cm^−1^ and 1035 cm^−1^ by the aromatic ether group [34,39,40].

After aging, significant changes were seen. After 120 h at 200 °C, some of the peaks were still recognizable in the spectrum, but after this the spectrum lost its shape. At 240 °C, the change already occurred after 120 h and no marked changes were seen after this. As with Rogers, strong wide peaks were formed in the carbonyl area, indicating oxidation. It was assumed that during the degradation process of the FR-4 its polymer chains were broken [39]. This affected its mechanical stability and caused it to lose its structure, as was seen in the samples after aging. It is likely that scission of chemical bonds also occurred in the Rogers material during aging. However, it had a higher level of inorganic substances and different structure from FR-4. This most likely made the Rogers more stable compared to FR-4.

### 3.2. Results from Real-Time Measurements

**Figure 5:**
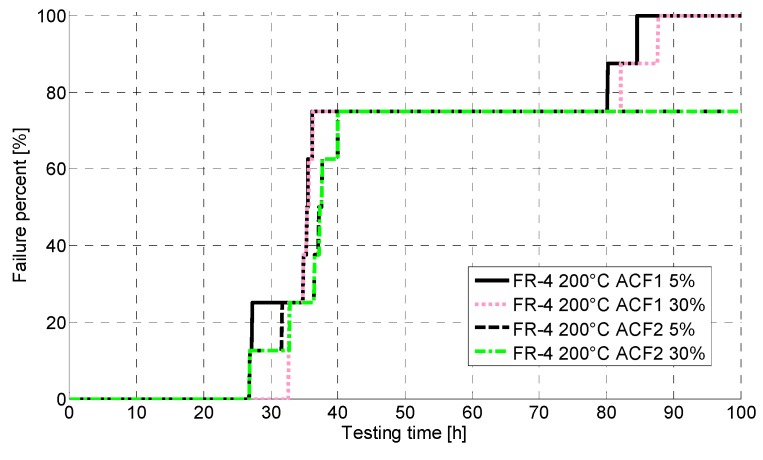
CDF for FR-4 at 200 °C.

Figure 5 shows the failures seen with the FR-4 PCB during testing at 200 °C with both failure criteria. No significant differences between different criteria were seen. The failures were observed to occur at a very early stage of the test. However, the samples were, for the most part, not fully broken, but fluctuating around the failure limit value for the whole testing time. No clear differences were seen between ACF1 and ACF2. ACF2, however, had one sample with lower failure criteria and two samples with higher failure criteria, which did not fail during testing but were observed to exceed the failure limits after testing when the temperature was decreased to ambient temperature.

**Figure 6:**
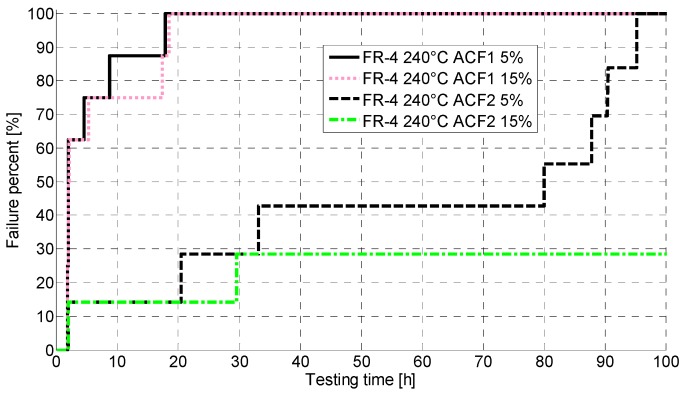
Cumulative distribution functions (CDFs) for FR-4 at 240 °C. ACF, Anisotropically conductive film.

Figure 6 shows the failures during the first 100 h for the FR-4 PCB during testing at 240 °C with both failure criteria. For ACF1 no significant differences were seen between the criteria. All the samples had failed before 20 h of testing, and over 60% of the samples already before two hours. Two ACF1 samples were observed to have sudden decreases in their resistance at the beginning of the test indicating a short circuit. Both samples exceeded the lower failure limit before the decreasing resistance was observed.

ACF2 was clearly more reliable but also showed a vast scatter in the results (Figure 6). All the samples exceeded the lower failure limit before 100 h of aging. The upper failure limit was exceeded much more slowly, and two of the ACF2 samples were not observed to fail during testing according to the upper failure limit. However, when the temperature was decreased from testing temperature to room temperature, these two samples were observed to exceed the failure limit. Similar to the aging at 200 °C, the samples were, for the most part, not totally broken but fluctuating around the failure limit value for the whole testing time.

**Figure 7:**
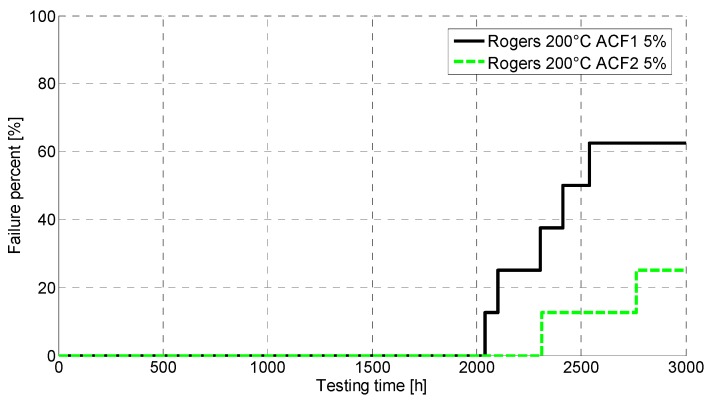
CDF for Rogers at 200 °C.

The reliability of the Rogers PCB was observed to be much better. Figure 7 presents the failures seen in aging at 200 °C. Over 60% of the ACF1 samples were seen to exceed the lower failure limit, however it occurred only after more than 2000 h of testing. However, only 25% of ACF2 samples exceeded the lower failure limit. When the higher failure limit was used, none of the ACF1 and ACF2 samples were observed to fail during 3000 h of aging. After testing, the resistance increases were observed to revert very close to the initial values.

**Figure 8:**
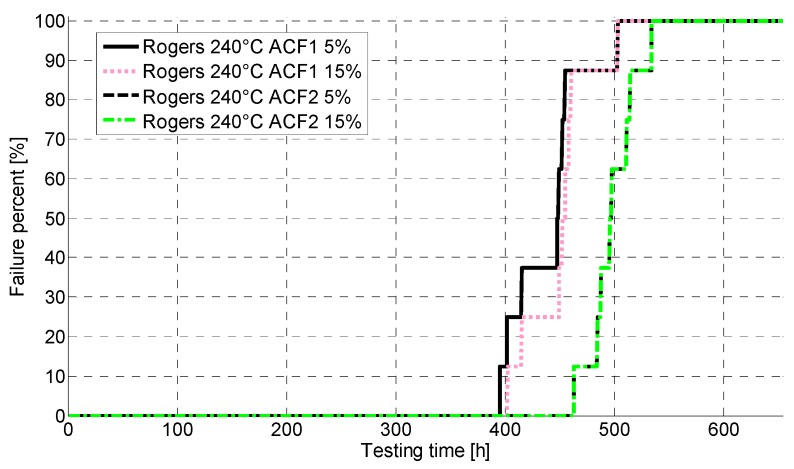
CDF for Rogers PCB at 240 °C.

Figure 8 presents the failures seen in the Rogers PCB in aging at 240 °C with both failure criteria. All samples were observed to fail between 400 h and 500 h. ACF2 was somewhat more reliable than ACF1. The failures with the different failure limits did not differ much from each other, especially with ACF2. The samples tended to fluctuate around the failure limit for approximately 30 h, after which open circuits were observed to form. The failures were not intermittent: after testing the resistance values remained high.

#### Failure Analysis

**Figure 9:**
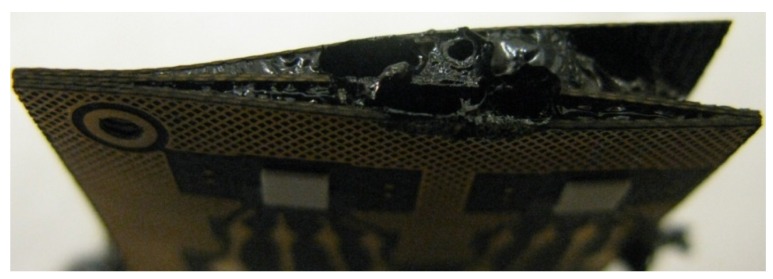
Inner layer after testing.

The electrical behavior of the FR-4 board was observed to be poor and unstable during testing. Clear changes in the FR-4 material could be seen during testing. Immediately after starting aging at both testing temperatures, the inner layer of the board reacted to the heat and started to degrade quickly, as shown in Figure 9. However, as all the functionality of the board was on the top layer, the samples did not necessarily fail due to the degradation of the inner layer.

**Figure 10:**
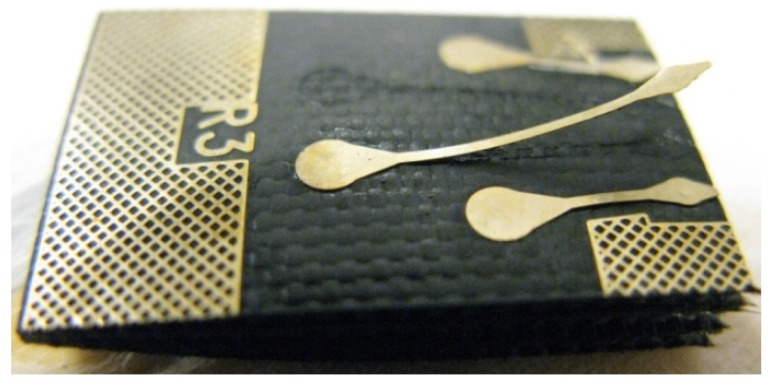
Detachment of the measurement wires in the corroded FR-4.

**Figure 11:**
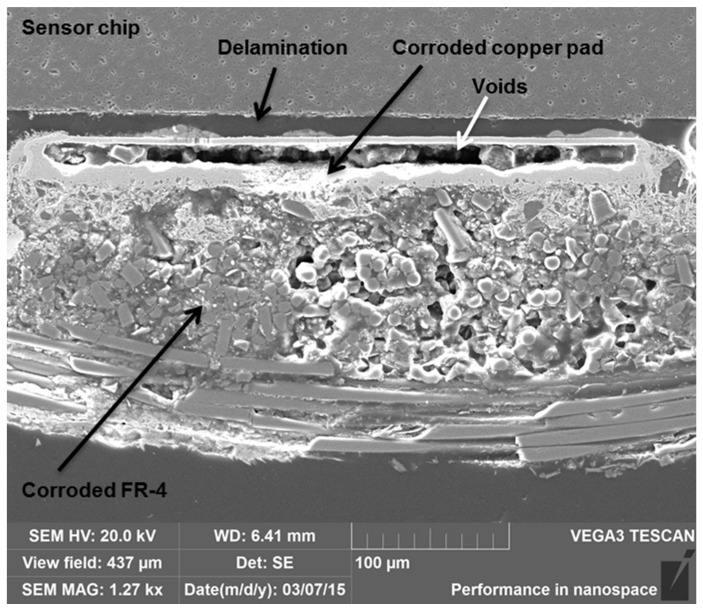
SEM Cross-section of a FR-4 sample aged for 40 days at 200 °C.

Additionally, the top layer of the FR-4 board corroded severely during the aging and clearly impaired the adhesion of copper traces to the board material (Figure 10). Therefore, the reliability of the measurement wires and their connections also markedly decreased. Severe corrosion of the substrate and interconnection materials, delamination between the sensor chip and the copper pads, as well as large voids in the pads were seen within the samples after short aging times (Figure 11). Consequently, the stability of the FR-4 material was observed to be poor even for short-duration high-temperature applications.

**Figure 12:**
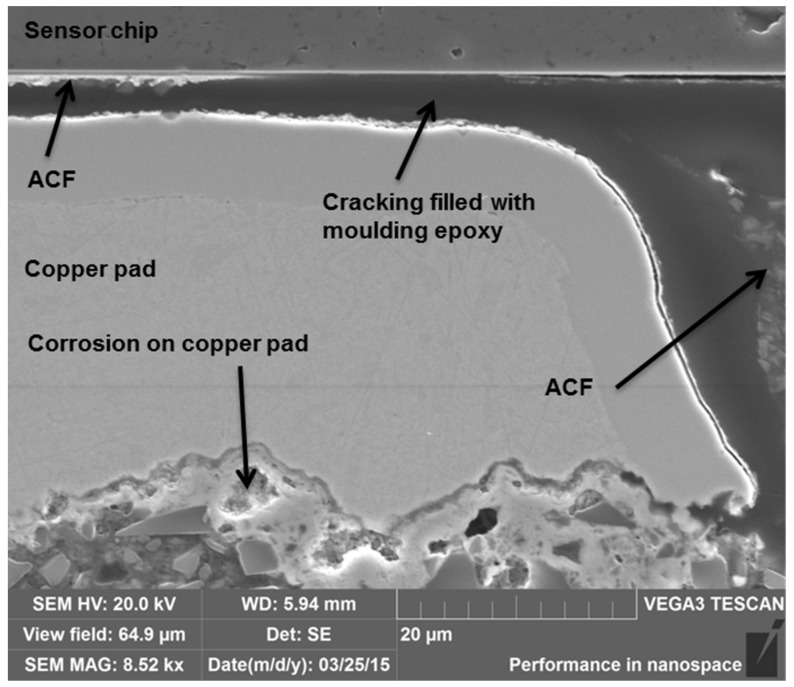
SEM image showing cracking filled with molding epoxy in a sample after 60 h of aging at 240 °C.

The electrical reliability of the Rogers PCB samples was found to be good, especially at 200 °C. However, the samples were observed to become mechanically fragile due to aging. Delamination and cracking between the sensor chip and the copper pads were seen in all the cross-sectioned samples, already occurring after 480 h of aging at 200 °C, even though the samples did not exceed the upper failure limit even after 3000 h of testing. The samples aged at 240 °C showed delamination and cracking after 22 h, again much earlier than the occurrence of the electrical failures. The delaminated parts were seen to been filled with molding epoxy, which indicated that delamination had likely occurred during the sample preparation for the cross-sectioning (Figure 12). This was a clear indication of the fragility of the samples, which would cause reliability problems in actual use conditions of the sensor components. However, the delamination and cracking seen with the Rogers PCBs were less than that seen with similar attachments made with a polyimide substrate, showing the importance of the substrate material for reliability [29,30].

During aging, the copper pads of the Rogers were observed to corrode. The first signs of corrosion at 200 °C were seen after 960 h of aging and after 60 h of aging at 240 °C. Additionally, a lot of pure copper was still seen even after 2400 h of aging at 200 °C. Compared to the identical samples with PI, the Rogers PCBs had less copper corrosion and it started to form later [29,30]. With the Rogers PCB, at both temperatures the corrosion started from the bottom of the pad at the interface between the substrate material and the copper pad (Figure 12).

**Figure 13:**
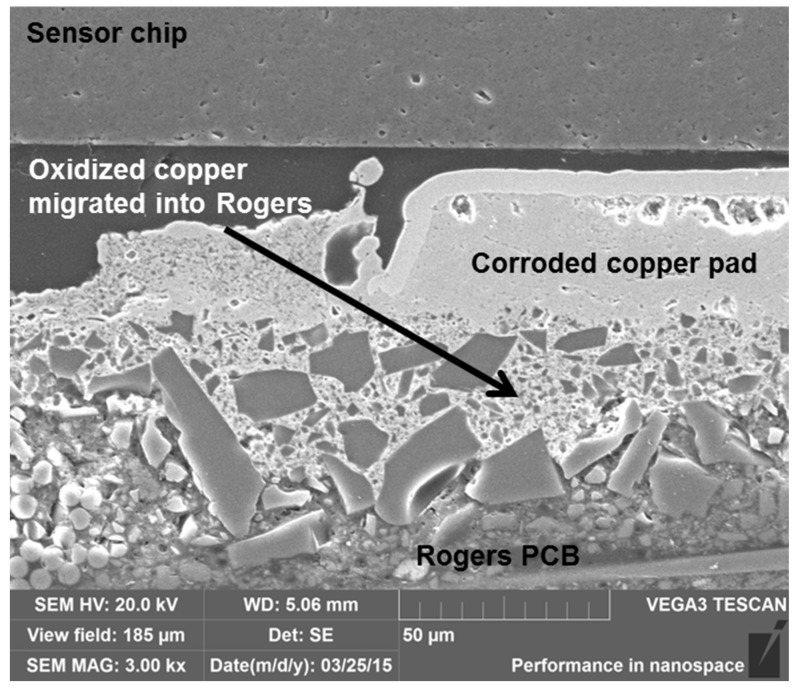
SEM migrograph showing the interface between Rogers and copper pad, indicating that the oxidized copper migrated into the Rogers material in a sample after 500 h of aging at 240 °C.

Reaction between the copper and the substrate material was also seen (Figure 13), and the oxidized material migrated within the structure. Similar diffusion of copper was seen in the PI substrates during aging [29,30]. When the diffusion of copper was compared between the Rogers PCB and PI PCB samples, the diffusion started slightly earlier in the Rogers PCB but proceeded faster in the PI PCB at both aging temperatures.

**Figure 14:**
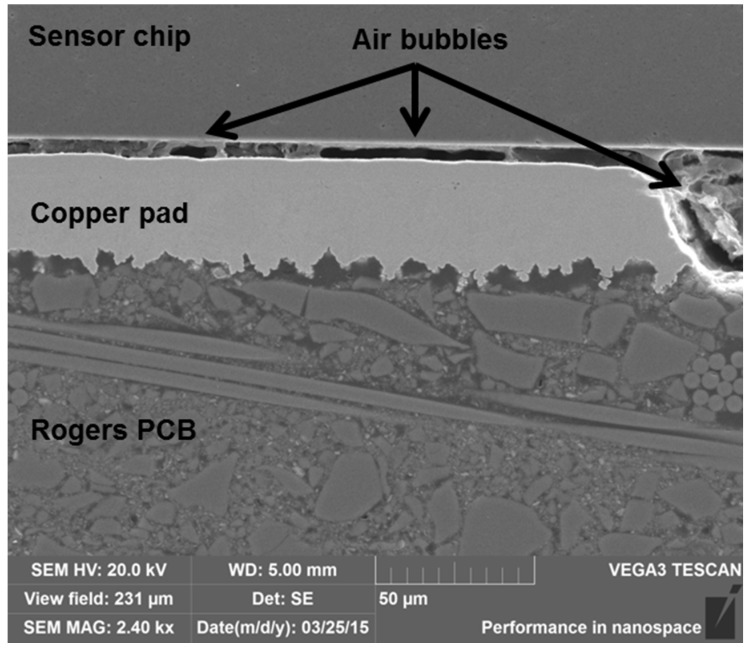
SEM micrograph showing air bubbles in the Rogers sample after 22 h of aging at 240 °C.

In addition to cracking and oxidation, the formation of air bubbles was seen in some of the samples at both aging temperatures. An example of the air bubbles is shown in Figure 14. Such air bubbles may have formed due to the degradation of the adhesive and the PCB and further reduced the mechanical stability of the interconnections.

### 3.3. Shear Testing

**Figure 15:**
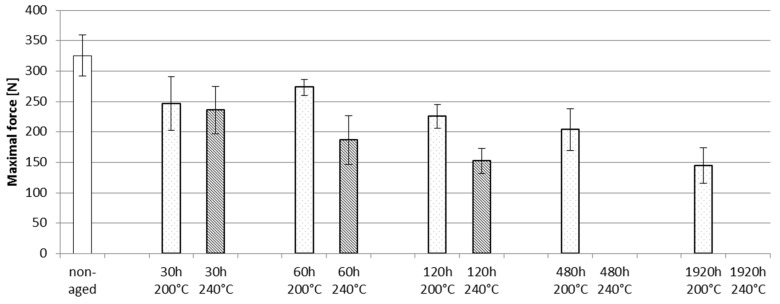
Shear testing results for Rogers samples.

The maximal force values of the shear testing are shown in Figure 15. Standard deviations are shown with error bars. Non-aged samples had the highest force values and the aging decreased the maximal forces. The force values were already seen to drop within a short aging time, as the force values for the samples aged for 30 h had lower maximal force values (approximately 246 N at 200 °C and 236 N at 240 °C) than the non-aged samples (approximately 325 N). Clear differences between the aging temperatures were seen after 60 h. At 200 °C, the force values decreased slowly, and after 1920 h of aging the average maximal force had decreased to 145 N. At 240 °C, the decrease occurred sooner. After 120 h of aging at 240 °C, the average maximum force value was on the same level as for the samples aged for 1920 h at 200 °C. After 480 h of aging at 240 °C, the interconnection was very fragile and the shear testing equipment was unable to record a force value for the testing. This was also approximately the point at which the electrical failures occurred.

**Figure 16:**
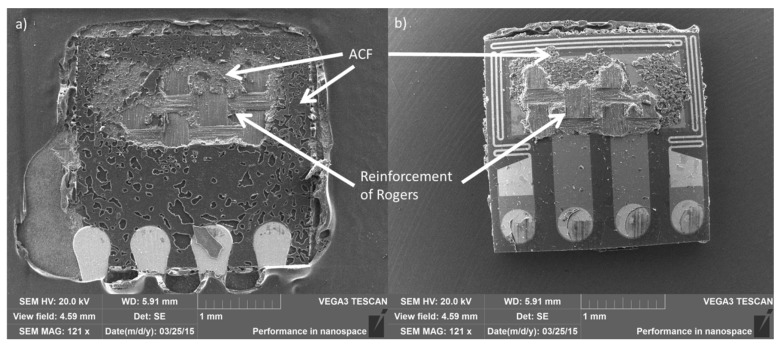
SEM images showing the fracture surface of (**a**) Rogers PCB; (**b**) chip aged for 120 h at 200 °C.

Failure analysis was done with the help of an optical microscope and SEM by observing the fracture surfaces on the sensor chip and Rogers PCB. The failure analysis revealed that the fracture occurred partially at the interface between the adhesive and the sensor (adhesion failure), partially within the adhesive (cohesion failure), and partially on the reinforcement layer of the PCB. At 200 °C this was the case for all samples, regardless of aging time (Figure 16). This suggests that all the materials were aging together and no weak links were found.

**Figure 17:**
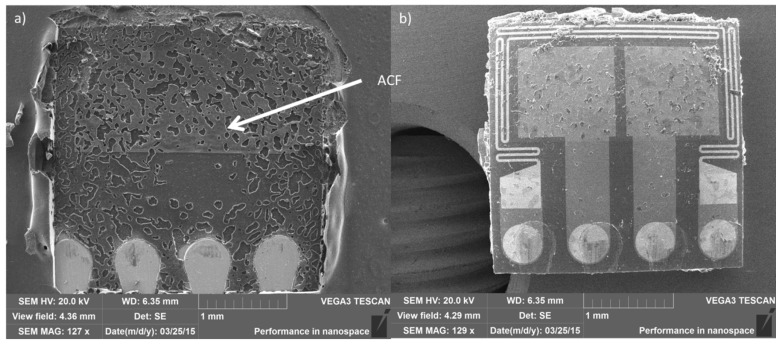
SEM images showing the fracture surface of (**a**) Rogers PCB; (**b**) chip aged for 120 h at 240 °C.

**Figure 18:**
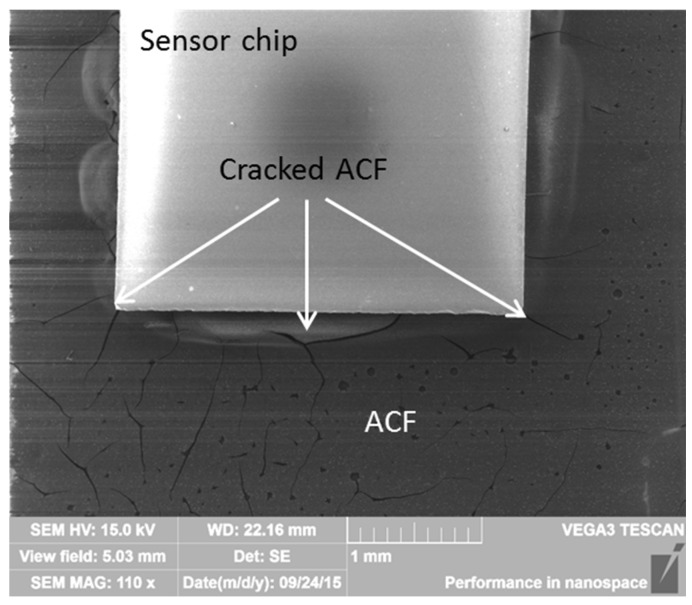
SEM image showing the cracked ACF surface of Rogers PCB sample aged for 480 h at 240 °C.

However, at 240 °C, the behavior was different. For the samples tested for 30 h at 240 °C, the fracture surface was similar to that of the samples aged at 200 °C, but the samples tested for 60 h and 120 h showed almost pure adhesion failures (Figure 17). The results from our earlier study showed that at 240 °C, the ACF degrades considerably faster than at 200 °C [30], and the results from the shear tests supported this result. The samples aged at 240 °C for 480 h and 1920 h were too fragile for shear testing. The adhesive next to the chip was observed to have cracked during aging (Figure 18).

## 4. Discussion

Very good high-temperature reliability was achieved in this study using stable PCB material. When the samples with Rogers PCB were aged at 200 °C, the first failure occurred after 2000 h. Shear testing also indicated that there was no weak link in the degradation of the materials, as the fracture surfaces did not change during aging. The Rogers samples were, however, observed to become mechanically brittle, which may be an important reliability issue in many use environments. Degradation could be seen in the DSC and FTIR analyses. However, it was observed that the degradation occurred at the start of the exposure to elevated temperatures, after which no further changes were seen, and the Rogers PCB remained stable. Furthermore, reliability at 240 °C was also good, as failures with the Rogers PCB were seen after 400 h of aging. Even though degradation of ACF material at 240 °C has been shown to start after 30 h of aging [30], the electrical failures in this study occurred hundreds of hours later. However, shear testing showed that the location of the fracture surface changed between 30 h and 60 h of exposure to pure adhesion failure, indicating a drastic impairment of the ACF at this temperature rather quickly.

The ACFs used differed from each other only in their particle type. ACF2 with nickel particles was observed to be somewhat more reliable than ACF1 with polymer particles. Similar results have been seen earlier with a similar test structure on PI PCB [29,30,31]. It is likely that at high temperatures the polymer particles also degrade, whereas nickel particles are more stable. This most likely explains the differences seen between the ACFs. On the other hand, polymer particles have been reported to withstand mechanical stresses better than rigid nickel particles [41]. However, if the high temperature caused degradation of the particles, their flexibility most likely suffered and consequently caused them to be more vulnerable to stresses in the structure.

**Figure 19:**
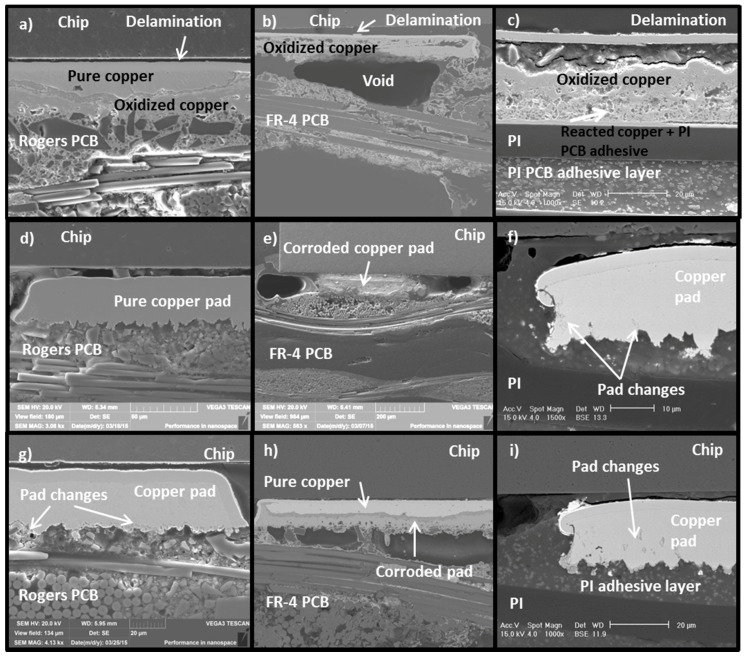
SEM micrographs of different PCBs exposed to varying temperature treatments. Top row: Cross-section after 3000 h at 200 °C for (**a**) Rogers PCB; (**b**) FR-4 PCB; (**c**) PI PCB. Middle row: Cross-section after 480 h at 200 °C for (**d**) Rogers PCB; (**e**) FR-4 PCB; (**f**) PI PCB. Bottom row: Cross-section after 60 h at 240 °C for (**g**) Rogers PCB; (**h**) FR-4 PCB; (**i**) PI PCB.

The fragility of the test samples after long term exposure was found to cause delamination and cracking in the samples after testing. However, severe corrosion was also seen in the test samples. The degree of corrosion observed in the test samples differed between the PCB materials. Figure 19 shows cross-sections for Rogers (Figure 19a) and FR-4 (Figure 19b) PCBs tested for 3000 h at 200 °C. Additionally, a cross-section of the PI PCB tested for the same time in a similar test is shown in Figure 19c. It can also be seen that delamination is more severe with the FR-4 and PI PCBs than with the Rogers PCB. Moreover, there is still some pure copper left after 3000 h of testing in the Rogers PCB but none in the FR-4 or PI PCBs. At 200 °C, the corrosion also started later for the Rogers PCB than for the PI and FR-4 PCBs (Figure 19d–f). The thickness of the copper varied between PCB materials.

After 480 h at 200 °C, no signs of corrosion were seen on the Rogers PCB samples, but PI PCB already had small changes at the corners of the copper pads [29]. FR-4, on the other hand, was already totally corroded after 480 h. At 240 °C, the corrosion occurred faster. Again, FR-4 corroded much faster than the Rogers and PI PCBs, but the differences between the Rogers and PI PCBs were smaller than at 200 °C. After 60 h of aging at 240 °C, the Rogers PCB started to corrode from the bottom of the pad, whereas the PI PCB only had pad changes elsewhere on the copper pad (Figure 19g–i). Copper changes seemed to start earlier with the PI PCB than with the Rogers, but proceeded faster in the Rogers than in the PI. Consequently, the Rogers PCB also achieved the best behavior in terms of corrosion. The results clearly showed that the substrate material is important for the reliability and behavior of interconnections at high temperatures, not only from the mechanical point of view but also in regard to the corrosion behavior.

## 5. Conclusions

In this study the performance and reliability of sensor attachments assembled on Rogers and FR-4 PCBs were tested in thermal storage tests at 200 °C and 240 °C. In addition to studying the electrical behavior of the samples, several material characterization techniques were used to analyze the PCB materials before and after aging. The mechanical performance was also studied with shear testing.

According to TGA and DTA analyses for non-aged samples, both PCB materials should have been stable at the aging temperatures, even though signs of oxidation were seen, because the weights of the samples already increased at the beginning of the measurement runs. When the aged PCB materials were studied with DSC and FTIR, clear degradation was seen, especially in the FR-4. FTIR also showed degradation of the Rogers, but fewer changes were seen with DSC. It was, however, observed that the degradation of the Rogers seen with FTIR occurred during the first 120 h of aging at both aging temperatures, after which no further changes were seen. Thus, the FR-4 was much less stable than the Rogers at high temperatures.

Furthermore, the electrical reliability of the FR-4 was poor. At 200 °C, the failures started to occur around 30 h of aging, whereas at 240 °C the failure free time was only a few hours. With the Rogers, much better reliability was achieved: at 200 °C, the failure free time was over 2000 h, and even at 240 °C, the first failures occurred around 400 h of aging. ACF2 with nickel particles achieved better performance. Failure analysis revealed fragility of the samples as delamination and cracking were seen. Oxidation of the materials was also seen, and the PCB material was observed to have an effect on the oxidation. The corrosion seen was especially severe in the FR-4 PCB. Shear testing for the Rogers showed that the mechanical reliability at 200 °C was quite good and no drastic drops in shear force values were seen. At 240 °C, the shear force decreased much faster and the ACF was observed to have degraded, as pure adhesion failure was seen after 60 h of aging.

All polymer materials were seen to affect the high temperature reliability of the sensor structure studied. PCB choice was shown to play a crucial role. ACFs showed very promising behavior with stable PCB material at very high temperatures, especially at 200 °C. However, at 240 °C, the materials were observed to degrade very quickly, even though electrically they were functional for several hundred hours.
